# Functional Polymorphisms in *IL13* Are Protective against High *Schistosoma mansoni* Infection Intensity in a Brazilian Population

**DOI:** 10.1371/journal.pone.0035863

**Published:** 2012-05-04

**Authors:** Audrey V. Grant, Maria Ilma Araujo, Eduardo Vieira Ponte, Ricardo Riccio Oliveira, Peisong Gao, Alvaro A. Cruz, Kathleen C. Barnes, Terri H. Beaty

**Affiliations:** 1 Department of Epidemiology, Bloomberg School of Public Health, The Johns Hopkins University, Baltimore, Maryland, United States of America; 2 Division of Allergy and Clinical Immunology, Department of Medicine, The Johns Hopkins University, Baltimore, Maryland, United States of America; 3 Serviço de Imunologia, Hospital Universitário Professor Edgard Santos, Salvador, Bahia, Brazil; 4 Programa para o Controle da Asma e Rinite Alérgica na Bahia, Federal University of Bahia School of Medicine, Salvador, Bahia, Brazil; University of Sao Paulo, Brazil

## Abstract

**Background:**

IL-13 is a signature cytokine of the helper T cell type 2 (TH2) pathway which underlies host defense to helminthic infection and activates production of IgE in both parasitized populations and in urban settings after allergen exposure.

**Methodology/Principal Findings:**

Two functional polymorphisms in *IL13*, rs1800925 (or c.1-1111C>T) and rs20541 (or R130Q) were previously found to be associated with *Schistosoma hematobium* infection intensity. They have not been thoroughly explored in *S. mansoni*-endemic populations, however, and were selected along with 5 tagging SNPs for genotyping in 812 individuals in 318 nuclear families from a schistosomiasis-endemic area of Conde, Bahia, in Brazil. Regression models using GEE to account for family membership and family-based quantitative transmission disequilibrium tests (QTDT) were used to evaluate associations with total serum IgE (tIgE) levels and *S. mansoni* fecal egg counts adjusted for non-genetic covariates. We identified a protective effect for the T allele at rs20541 (P = 0.005) against high *S. mansoni* egg counts, corroborated by QTDT (P = 0.014). Our findings also suggested evidence for protective effects for the T allele at rs1800925 and A allele at rs2066960 after GEE analysis only (P = 0.050, 0.0002).

**Conclusions/Significance:**

The two functional variants in *IL13* are protective against high *S. mansoni* egg counts. These markers showed no evidence of association with tIgE levels, unlike tIgE levels previously studied in non-parasitized or atopic study populations.

## Introduction

The worldwide prevalence of schistosomiasis is high at 200 million infected individuals, creating a substantial public health burden. [1] Schistosomiasis occurs in areas where humans come into contact with water harboring the intermediate snail host for *Schistosoma mansoni* in parts of South America, Africa and the Middle East; *S. haematobium* in Africa and the Middle East; or *S. japonicum* in China, South-East Asia and the Philippines. [1] Infection occurs when cercariae burrow directly through the skin, maturing into the adult form in the portal vasculature. Females lay eggs which traverse into the intestine (*S. mansoni* and *S. japonicum*) or the bladder (*S. haematobium*). [2] Control of schistosomiasis is cumbersome, and reinfection after treatment is common. [1] These obstacles have motivated research to better understand schistosomiasis host immunity to identify those individuals susceptible to greatest intensity of infection for targeted treatment and prevention. [3], [4].

Host immune response to infection involves differentiation of helper T cells into two major subtypes known as TH1 and TH2 cells. While TH1 cells elicit cellular immunity against intracellular bacteria and viruses, infection by helminths (including *S. mansoni*) induces a TH2 humoral response, accompanied by release of the cytokines IL-4, IL-5 and IL-13. Although effector mechanisms of TH2 activation in parasitic disease are not fully understood, these result in acquired immunity through antibody-dependent cellular cytotoxicity mediated by production of IgE antibodies and eosinophils. [2], [5] Activation of TH2 immunity also underlies atopic diseases, including asthma, in response to stimulation by innocuous allergens. The cytokine IL-13 is involved in immunoglobulin class switching to immunoglobulin E (IgE) and in airway hypersensitivity, mucus hypersecretion, and inflammation of the bowel. [6], [7] IgE production is thought to have evolved as a protective feature in host defense against helminthes [2], but total serum IgE (tIgE) levels have been widely studied as a surrogate endophenotype relating to atopic disease [8], although it is seldom studied in the context of helminthic infection.

Considerable evidence points to a genetic basis for variation in tIgE levels in urban populations [9], [10] and we have previously reported high heritability for tIgE levels in a schistosomiasis-endemic Brazilian population, as well as for burden of infection by *S. mansoni,* measured by fecal egg counts. [11] To explore specific genetic factors underlying this heritability, we focused on variation in *IL13* located in the 5q31-q33 region. Linkage studies have identified the 5q31-q33 region as a locus influencing tIgE levels in populations of high-income countries [12], as well as intensity of parasite infection in Brazilian [13] and Senegalese [14] schistosomiasis-endemic populations. In terms of specific variants, the T allele at the promoter polymorphism rs1800925 (or c.1-1111C>T) [8] and the T allele at the non-synonymous coding variant rs20541 [8] (or R130Q where the T allele creates an amino acid change Arg130 to Gln130) in *IL13* have repeatedly been found to be associated with high tIgE levels, high IgE levels specific to allergens and atopic phenotypes, with strongest associations to tIgE levels reported among non-atopic individuals of European ancestry. Functional studies have demonstrated increased binding of nuclear proteins to the *IL13* promoter region when the T allele at rs1800925 was present. [15], [16] IL-13 containing the variant Gln residue was more active than the Arg form, and serum levels of IL-13 were higher in IL-13Gln-bearing individuals. [17] Thus, both variants increase amount or activity of IL-13 and as expected are associated with higher tIgE levels. These variants in *IL13* have also been explored in schistosomiasis-endemic populations. In particular, rs1800925 and rs20541 were protective against high *S. hematobium* infection intensity [18] and rs1800925 against high *S. mansoni* intensity, [19] for alleles associated with elevated tIgE levels in urban setting studies.

We investigated associations between these and other variants in *IL13,* covering the full gene (including rs1800925 and rs20541), for two quantitative traits (tIgE levels and *S. mansoni* egg counts) in a Brazilian population endemic for schistosomiasis. *IL13* variants have not been previously tested for association with tIgE levels in a parasitized population. Measured tIgE levels represent activation of TH2 immunity largely in response to infection by helminths, and *S. mansoni* egg counts (corresponding to worm burden) represent the impact of host immunity or overall effectiveness of schistosomiasis host immunity (including TH2 activation and effector mechanisms). Therefore we were able to investigate the influence of *IL13* variation on two key aspects of *S. mansoni* host immunity.

## Methods

### Ethics Statement

The research protocol was approved by Institutional Review Boards (IRBs) at Johns Hopkins University School of Medicine and the Federal University of Bahia and was endorsed by the National Commission for Ethics in Human Research in Brazil. In accord with the protocol, all subjects enrolled in the study gave written consent when possible or oral consent in the case of subjects unable to read or to provide a written signature. The protocol for providing consent thus covered the full target population which includes some individuals who are literate and others who are illiterate. Children gave their assent, and a parent or a legal guardian provided written or oral consent. Oral consent was documented by a witness able to provide a written signature on a separate line incorporated into the consent form for this purpose specifically approved by the IRBs at Johns Hopkins University School of Medicine and the Federal University of Bahia.

### Study Design and Clinical Characteristics

This study was performed on a Brazilian study population from a schistosomias-endemic area of Conde, Bahia conducted between July and September 2004 (as described previously). [11] A total of 812 subjects were enrolled based on a whole-population ascertainment scheme, comprising two large families of 535 and 310 individuals each, 38 families with three to 36 members, and 44 singletons. These pedigrees were broken down into 318 nuclear families, and connecting individuals were duplicated when performing family-based association tests, or assigned to one family for GEE regression tests. *S. mansoni* egg counts on 397 individuals from 3–5 stool samples per individual were obtained using the Kato-Katz method [20], [21] and egg counts means were calculated for all available samples, while presence of *Ascaris lumbricoides*, *Trichuris trichiura*, and hookworm eggs was also measured. Total serum IgE levels were measured on 572 individuals using chemiluminescence (ADVIA Centaur Bayer Corporation) in Salvador, Brazil. The raw values of tIgE levels were adjusted for non-genetic covariates (sex, age, smoking history, and infection by other helminths) and *S. mansoni* egg counts were adjusted for sex, age, and exposure to infested water, and tests of genetic association were conducted on residuals.

### SNP Selection

In addition to the two functional polymorphisms rs1800925 and rs20541, coverage of the full gene was assured based on detailed evaluation of linkage disequilibrium from an *IL13* sequencing study. Tarazona-Santos and Tishkoff identified five haplotype tagging single nucleotide polymorphisms (SNPs) in *IL13* through sequencing based on West African, South American (with a strong Amerindian component) and European populations. [22] Thus, to best reflect the admixed Brazilian study population (derived from West African, European and Amerindian ancestral populations, with substantial African ancestry in the state of Bahia [23]), these five additional SNPs covering the gene were selected for genotyping, bringing the total to 7 SNPs. SNPs with a minor allele frequency (MAF) of 10% or below were excluded. Linkage disequilibrium (LD) bins were defined as groups of SNPs with pairwise r^2^>0.8. Genotyping of these 7 SNPs was conducted using the TaqMan probe-based 5′ nuclease assays (Applied Biosystems, Foster City, CA, USA). If an assay failed on a sample, this was repeated up to three times.

### Statistical Methods

As described previously, the two quantitative traits were initially log-10 transformed and adjusted for non-genetic covariates so genetic effects on remaining variation could be assessed. Thus, log10-transformed values of tIgE levels were adjusted for age, sex, smoking status, household smoking exposure and helminthic infection status using STATA 8.2 (StataCorp. StataCorp LP. College Station, TX). [11] Similarly, log-10 transformed *S. mansoni* egg counts were adjusted for age, sex and four categories of exposure to infested water. [11] Associations between the 7 selected markers and the two quantitative traits were tested under an additive model, which has been shown to perform well even when the true genetic model is not additive (i.e. dominant or recessive) using two approaches, first regression-based GEE methodology which considers the nuclear family as a cluster, [24] and second using variance components models implemented in QTDT (quantitative transmission disequilibrium tests; v.4.6 http://www.sph.umich.edu/csg/abecasis/QTDT/ [25], [26] which considers transmissions within and between families. GEE regression allows each subject with genotype and phenotype data to contribute to the association test while taking into account correlations within a nuclear family. The QTDT statistic tests whether the trait mean in offspring differs according to transmission of a target marker allele to the child from a heterozygous parent. [26] A maximum likelihood based test of association for individual environment and polygenic genetic components was applied testing both association and transmission disequilibrium for each marker. In this approach, the phenotypic variance is partitioned into between- and within-family components. The family-based QTDT is robust to potential confounding due to population stratification, which is a particular advantage in the highly admixed Brazilian study population. QTDT software does not allow for estimation of haplotypic associations. Associations with the two functional SNPs were evaluated at a nominal α = 0.05 because these markers were selected under a strong *a priori* hypothesis, and tagging SNPs were evaluated at a more stringent α = 0.003 (rounded after application of the Bonferroni correction for 14 tests; 7 SNPs * 2 test statistics).

## Results

Clinical characteristics for the 812 subjects, including 222 founders are described in detail elsewhere. [11], [27] There were fewer male than female participants (44.2% males), and the mean age of the population was 27 years. The proportion of subjects infected by any helminth was 83.5%, the proportion infected with *S. mansoni* was 48.9%. Geometric means for all subjects, including infected and uninfected individuals, for adjusted tIgE levels and *S. mansoni* egg counts were 2511.9 ng/mL (Standard deviation (SD): 2.7; range: 4.5 to 28020 ng/mL) and 15.5 count/g fecal matter (SD: 6.5; range: 0 to 1579 count/g fecal matter), respectively.

Genotyping call rates for the 7 *IL13* SNPs ranged from 90 to 99%. Mendelian inconsistencies were identified using Sib-Pair v.1.00a17 (http://www.qimr.edu.au/davidD/), and underlying genotypes by SNP and nuclear family were set as missing. All 7 SNPs were found to be in Hardy-Weinberg equilibrium among unrelated founders (P>0.001). The final set of 7 SNPs with allele frequencies are displayed in Table 1. All but two markers had a MAF >0.1.

**Table 1:** The 7 *IL13* SNPs among 222 founders in the Brazilian study sample.

Marker	Chr	Chr Position	FunctionalPosition	Allele	MAF
**rs1800925**	**5**	**132020708**	**5′ UTR**	**C/T**	**0.313**
rs2069743	5	132021174	5′ UTR	A/G	0.098
rs2066960	5	132022334	Intron 1	C/A	0.202
rs1295686	5	132023742	Intron 3	G/A	0.449
**rs20541**	**5**	**132023863**	**Exon 4**	**C/T**	**0.206**
rs1295685	5	132024344	Exon 4	C/T	0.144
rs2069750	5	132024496	Exon 4	G/C	0.098

Abbreviations: SNP, single-nucleotide polymorphism; UTR, untranslated region; MAF, minor allele frequency; Chr, chromosome. Chromosomal position according to db126; major and minor alleles in Brazilian data before and after forward slash, respectively; ancestral allele according to db126 data underlined. Atopy-related SNPs are indicated in bold and tagging SNPs that are not atopy-related are in regular font.

**Figure 1:**
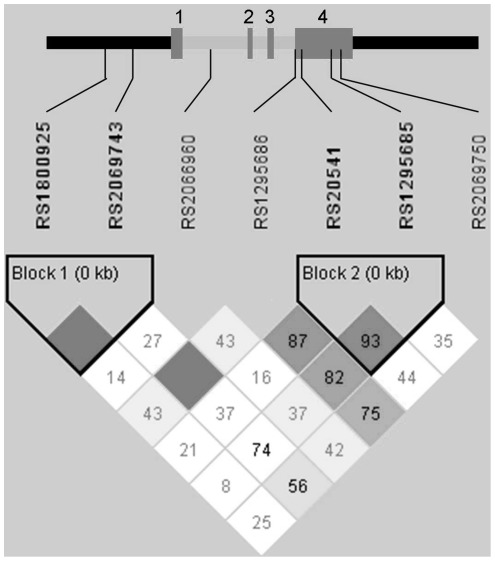
Pairwise linkage disequilibrium (LD) within Haploview using the D’ statistic for *IL13*. Intensity of shading indicates the degree of confidence in the D’ value. Dark filled squares indicate a D’ value of 1. Untranslated regions are indicated with black bars, exons with dark gray bars and introns with light gray bars. Exons are numbered from 5′ to 3′.

Pairwise LD based on the D’ statistic was calculated using HaploView (http://www-genome.wi.mit.edu/personal/jcbarret/haplo) among founders as shown in Figure 1. LD blocks were defined using Gabriel et al.’s algorithm, and there were two LD blocks, one in the promoter region and one in exon 4. [28].

Table 2 summarizes GEE regression analyses of residual tIgE levels and *S. mansoni* egg counts on genotype, after adjustment for non-genetic factors. QTDT association test results are displayed in Table 3. Alleles T, A and T at the three SNPs (rs1800925, rs2066960, and rs20541 respectively, spanning the promoter to the exon 4 region) showed evidence of association with low levels of *S. mansoni* egg counts (P = 0.05, 0.0002, and 0.005, respectively) based on GEE regression (Table 2). For these SNPs, the major allele increased risk while the minor allele was protective against high burden of infection. Association of *S. mansoni* egg counts with the non-synonymous coding SNP rs20541 was supported by QTDT analysis (P = 0.014). Geometric means were 7.1, 13.4 and 17.5 count/g fecal matter for individuals with the TT, CT and CC genotypes respectively at rs20541. The promoter SNP rs1800925 showed the same direction of association for GEE regression, although without statistical significance (Table 3). No associations were found for tIgE levels, however. This absence of association with tIgE levels is unlikely to merely result from low statistical power as our previous work on the same Brazilian study population, similarly investigating variants in the promoter region in a TH2-pathway cytokine gene, identified statistically significant associations between atopy-related SNPs in *IL10* and tIgE levels. [27] Marker rs2066960 located within intron 1 displayed relatively low pairwise LD (D’ from 14 to 43%) with other genotyped SNPs, suggesting this signal is likely to be independent although replication will be required to confirm this finding.

**Table 2:** Generalized Estimating Equation association tests for individual SNPs with tIgE levels (N = 572) and *Schistosoma mansoni* egg counts (N = 397) under the additive model.

Marker	Allele	tIgE^a^		*S.m.* egg count^b^	
		P^c^	Dir^c^	P^c^	Dir^c^
**rs1800925**	**T**	**0.408**	**neg**	**0.050** d	**neg**
rs2069743	G	0.351	neg	0.084	neg
rs2066960	A	0.066	pos	0.0002^d^	neg
rs1295686	A	0.045	pos	0.706	neg
**rs20541**	**T**	**0.779**	**neg**	**0.005** d	**neg**
rs1295685	T	0.157	neg	0.014	neg
rs2069750	C	0.473	pos	0.392	neg

Abbreviations: Dir, direction of association; pos, positive; neg, negative.

a log(10)-transformed values adjusted for age, sex, smoking, and helminthic infection status.

b log(10)-transformed values adjusted for age, sex, and low, medium and high exposure to infested water sources.

c P-values and direction of association with high values are given for the beta-coefficient for the minor allele under the additive model.

d P-values are statistically significant with α = 0.05 for atopy-related SNPs (bold) and α = 0.005 for tagging SNPs that are not atopy-related (unbolded).

**Table 3:** QTDT association results for individual *IL13* SNPs with tIgE levels and *Schistosoma mansoni* egg count.

Marker	Allele	tIgE^a^				*S.m.* egg count^b^			
		χ^2^ c	P^c^	Dir^c^	N offspring	χ^2^ c	P^c^	Dir^c^	N offspring
**rs1800925**	**T**	**1.28**	**0.258**	**neg**	**247/686**	**0.09**	**0.768**	**neg**	**175/480**
rs2069743	G	0.27	0.605	neg	149/703	1.49	0.222	pos	108/485
rs2066960	A	0.82	0.365	pos	154/691	0.17	0.676	neg	99/481
rs1295686	A	0.02	0.876	pos	256/693	1.05	0.305	neg	188/480
**rs20541**	**T**	**0.22**	**0.639**	**neg**	**184/680**	**5.99**	**0.014** d	**neg**	**126/465**
rs1295685	T	0.02	0.882	neg	144/711	1.31	0.252	neg	93/488
rs2069750	C	0.54	0.463	neg	129/661	0.22	0.637	pos	104/445

Abbreviations: Dir, direction of association; pos, positive; neg, negative; N offspring, number of informative offspring over number of offspring evaluated; neg., negative; pos., positive; QTDT, quantitative transmission disequilibrium test; *S.m.* count, *Schistosoma mansoni* egg count per gram of fecal matter; SNP, single-nucleotide polymorphism; tIgE, total serum IgE.

a log(10)-transformed values adjusted for age, sex, smoking, and helminthic infection status.

b log(10)-transformed values adjusted for age, sex, and low, medium and high exposure to infested water sources.

c Likelihood ratio tests were compared to obtain tests of evidence of association in the presence of linkage for models where (1) means were considered as composed of covariate effects and between- and within-family components and (2) means were considered as composed of covariate effects and between-family components only. For both models, the variance was set to be composed of polygenic and individual environmental effects.

d P-values are statistically significant with α = 0.05 for atopy-related SNPs (bold) and α = 0.005 for tagging SNPs that are not atopy-related (unbolded).

## Discussion

Genetic associations observed for functional polymorphisms, rs1800925 and rs20541, which are protective against high *S. mansoni* egg counts in our Brazilian study population, support evidence for a protective effect against high infection intensity for alleles associated with atopic disease, and as previously observed in a population endemic for the *S. haematobium* species of schistosomes. For the SNP rs20541, the protective effect of the T allele was identified using both family-based QTDT analysis and GEE regression, while for the SNP rs1800925, statistical significance of the protective effect at the T allele was achieved using GEE regression only. Given the MAF for SNP rs1800925 in our study, statistical power for detecting the association using regression was substantial: it was close to 80% while power was under 40% for the 247 informative trios. [29] Thus, the lack of association using QTDT can be explained by insufficient power, and our finding of association for rs1800925 using GEE regression remains credible given higher statistical power inherent in regression-based methods for similar sample sizes. In the literature, in a Malian population infected with *S. haematobium,* the T allele at rs1800925 was protective against higher infection levels (P = 0.01), [18] and in a subsequent investigation of the promoter region, no other marker showed a greater protective effect within the full study population. [30] Also in this Malian study, in a family-based analysis, the T allele at rs20541 was found to be preferentially transmitted to offspring in the highest 10% of *S. hematobium* infection intensity. [18] A recent Kenyan study on a population infected with *S. mansoni* showed heterozygotes at rs1800925 were resistant to reinfection, but this finding specific to the heterozygous genotype class is biologically difficult to interpret and may reflect the small sample size. [19] Thus our study extends previous findings of association of the T allele at rs1800925 and the T allele at rs20541 with high *S. hematobium* infection intensity to high *S. mansoni* infection intensity.

From studies in industrialized populations, considerable evidence points to some influence by variants in the *IL13* promoter, in particular rs1800925 and rs20541, on risk of atopy among European and Asian ancestry cohorts. [8] The T allele at rs1800925 was associated with asthma and atopy among European Americans and Europeans, [31], [32] and the T allele at rs20541 was associated with bronchial asthma among Europeans. [33] The T alleles for both SNPs were the risk alleles for atopy, while we have shown these are protective against high burden of *S. mansoni* infection in this highly parasitized Brazilian study of an admixed population. Our data are consistent with an evolutionary hypothesis proposing the TH2 pathway developed, at least in part, as a mechanism to protect humans (and their ancestors) from helminthic infection, and certain genetic risk factors for atopic disease are a vestige of this selective process. [34].

The lack of association between *IL13* variants and tIgE levels in this Brazilian parasitized study population can be evaluated in the context of previous findings in industrialized populations where tIgE levels have been studied among different groups, such as atopic individuals who have been exposed to allergens, non-atopic individuals, or the general population. Specifically, in a linkage study identifying the 5q31-q33 region for tIgE levels, the signal was only present in the subset of the population among individuals where no IgE specific to antigens was detected. [35] Similarly, in genetic association studies focusing on *IL13* variants, associations have been identified for tIgE levels among non-atopic individuals or the general population, but rarely among atopic individuals. [8] For example, in the large British 1958 Birth Cohort following 4,570 individuals from the general population, basal tIgE means significantly differed between genotypes at both rs1800925 and rs20541. [36] The helminth-endemic setting of the present Brazilian study population meant tIgE levels reflected a mixture of IgE specific to *S. mansoni* and IgE specific to other helminths or antigens. Basal IgE levels without any antigenic stimulation, which are conditions under which we would expect to detect association with *IL13* polymorphisms based on the literature as discussed above, cannot be measured in a helminth-endemic setting by definition. Thus, the finding of no associations between *IL13* polymorphisms and tIgE levels in this Brazilian study is consistent with studies conducted in populations of industrialized communities.

In our Brazilian study population, the age profile for *S. mansoni* egg counts features a characteristic peak in infection intensity at 15 years, which represents acquisition of natural immunity to infection in adolescence shown previously. [11] For tIgE levels, a different pattern was observed and mean values were highest among young children aged 6–9 years, and then declined with increasing age. We therefore performed association analyses across SNPs on subsets of independent individuals from the full study population comprising children under 15 years and individuals over 15 years of age. No differences in evidence for association were observed in the two subsets (data not shown).

Thus, evaluating our data in the context of the literature suggests IL-13 acts most strongly on TH2 pathway effector mechanisms (as reflected in statistical associations with *S. mansoni* egg counts) rather than on TH2 pathway activation (as reflected by lack of association with tIgE levels). It is also possible IL-13 acts during only early TH2 activation in an antigen-specific manner, but IgE levels specific against schistosomiasis antigens were not available in this Brazilian study.

The two quantitative traits, tIgE levels, representing TH2 pathway activation, and *S. mansoni* egg counts, reflecting global impact of TH2 effector mechanisms and helminthic host immunity, provided a unique opportunity for genetic dissection of the TH2 pathway in the context of schistosomiasis endemicity. In summary, we have found significant associations between two well-known functional variants and *S. mansoni* egg counts, and a striking lack of association with tIgE levels. Since the functional effect of both variants on the gene product, IL-13, is to increase its amount or activity, this finding suggests IL-13 functions to increase anti-helminth immunity, and functional variants may be an evolutionary vestige of selective forces that result in atopic phenotypes in modern, industrialized settings. Our evaluation of *IL13* variation has demonstrated TH2 pathway genes shown to carry variants that impact on atopic disease are good candidates to evaluate determinants of host immunity to helminthic infections. Moreover, genetic association studies in schistosomiasis-endemic populations allow further fine dissection of the TH2 pathway and its role in disease, both helminthic and atopic.

## References

[1] King CH, Dickman K, Tisch DJ (2005). Reassessment of the cost of chronic helmintic infection: a meta-analysis of disability-related outcomes in endemic schistosomiasis.. Lancet;.

[2] Pearce EJ, MacDonald AS (2002). The immunobiology of schistosomiasis.. Nat Rev Immunol;.

[3] Bethony JM, Quinnell RJ (2008). Genetic epidemiology of human schistosomiasis in Brazil.. Acta Trop;.

[4] Quinnell RJ (2003). Genetics of susceptibility to human helminth infection.. Int J Parasitol;.

[5] Caldas IR, Campi-Azevedo AC, Oliveira LF, Silveira AM, Oliveira RC (2008). Human schistosomiasis mansoni: immune responses during acute and chronic phases of the infection.. Acta Trop;.

[6] Kelly-Welch AE, Hanson EM, Boothby MR, Keegan AD (2003). Interleukin-4 and interleukin-13 signaling connections maps.. Science;.

[7] Vercelli D (2002). Genetics of IL-13 and functional relevance of IL-13 variants.. Curr Opin Allergy Clin Immunol;.

[8] Ober C, Hoffjan S (2006). Asthma genetics 2006: the long and winding road to gene discovery Genes Immun;.

[9] Meyers DA, Beaty TH, Colyer CR, Marsh DG (1991). Genetics of total serum IgE levels: a regressive model approach to segregation analysis.. Genet Epidemiol;.

[10] Gerrard JW, Rao DC, Morton NE (1978). A genetic study of immunoglobulin E. Am J Hum Genet;.

[11] Grant AV, Araujo MI, Ponte EV, Oliveira RR, Cruz AA (2008). High heritability but uncertain mode of inheritance for total serum IgE level and Schistosoma mansoni infection intensity in a schistosomiasis-endemic Brazilian population.. J Infect Dis;.

[12] Hoffjan S, Ober C (2002). Present status on the genetic studies of asthma.. Curr Opin Immunol;.

[13] Marquet S, Abel L, Hillaire D, Dessein H, Kalil J (1996). Genetic localization of a locus controlling the intensity of infection by Schistosoma mansoni on chromosome 5q31-q33.. Nat Genet;.

[14] Muller-Myhsok B, Stelma FF, Guisse-Sow F, Muntau B, Thye T (1997). Further evidence suggesting the presence of a locus, on human chromosome 5q31-q33, influencing the intensity of infection with Schistosoma mansoni.. Am J Hum Genet;.

[15] van der Pouw Kraan TC, van Veen A, Boeije LC, van Tuyl SA, de Groot ER (1999). An IL-13 promoter polymorphism associated with increased risk of allergic asthma.. Genes Immun;.

[16] Cameron L, Webster RB, Strempel JM, Kiesler P, Kabesch M (2006). Th2 cell-selective enhancement of human IL13 transcription by IL13–1112C>T, a polymorphism associated with allergic inflammation.. J Immunol;.

[17] Arima K, Umeshita-Suyama R, Sakata Y, Akaiwa M, Mao XQ (2002). Upregulation of IL-13 concentration in vivo by the IL13 variant associated with bronchial asthma.. J Allergy Clin Immunol;.

[18] Kouriba B, Chevillard C, Bream JH, Argiro L, Dessein H (2005). Analysis of the 5q31-q33 locus shows an association between IL13–1055C/T IL-13–591A/G polymorphisms and Schistosoma haematobium infections.. J Immunol;.

[19] Gatlin MR, Black CL, Mwinzi PN, Secor WE, Karanja DM (2009). Association of the gene polymorphisms IFN-gamma +874, IL-13–1055 and IL-4–590 with patterns of reinfection with Schistosoma mansoni.. PLoS Negl Trop Dis;.

[20] Katz N, Zicker F, RS Rocha RS, VB Oiveira VB (1978). Reinfection of patients in schistosomiasis mansoni endemic areas after specific treatment.. Revista do Instituto de Medicina Tropical de Sao Paulo;.

[21] Katz N, Coelho PM, Pellegrino J (1970). Evaluation of Kato’s quantitative method through the recovery of Schistosoma mansoni eggs added to human feces.. Journal of Parasitology;.

[22] Tarazona-Santos E, Tishkoff SA (2005). Divergent patterns of linkage disequilibrium and haplotype structure across global populations at the interleukin-13 (IL13) locus.. Genes Immun;.

[23] Azevedo ES (1980). Subgroup studies of black admixture within a mixed population of Bahia, Brazil.. Ann Hum Genet; 44(Pt.

[24] Zeger SL, Liang KY (1986). Longitudinal data analysis for discrete and continuous outcomes.. Biometrics;.

[25] Abecasis GR, Cookson WO, Cardon LR (2000). Pedigree tests of transmission disequilibrium.. Eur J Hum Genet;.

[26] Abecasis GR, Cardon LR, Cookson WO (2000). A general test of association for quantitative traits in nuclear families.. Am J Hum Genet;.

[27] Grant AV, Araujo MI, Ponte EV, Oliveira RR, Cruz AA (2011). Polymorphisms in IL10 are associated with total Immunoglobulin E levels and Schistosoma mansoni infection intensity in a Brazilian population.. Genes Immun;.

[28] Gabriel SB, Schaffner SF, Nguyen H, Moore JM, Roy J (2002). The structure of haplotype blocks in the human genome.. Science;.

[29] Gauderman WJ (2002). Sample size requirements for matched case-control studies of gene-environment interaction.. Stat Med;.

[30] Isnard A, Kouriba B, Doumbo O, Chevillard C (2011). Association of rs7719175, located in the IL13 gene promoter, with Schistosoma haematobium infection levels and identification of a susceptibility haplotype.. Genes Immun;.

[31] Hummelshoj T, Bodtger U, Datta P, Malling HJ, Oturai A (2003). Association between an interleukin-13 promoter polymorphism and atopy.. Eur J Immunogenet;.

[32] Howard TD, Koppelman GH, Xu J, Zheng SL, Postma DS (2002). Gene-gene interaction in asthma: IL4RA and IL13 in a Dutch population with asthma.. Am J Hum Genet;.

[33] Heinzmann A, Mao XQ, Akaiwa M, Kreomer RT, Gao PS (2000). Genetic variants of IL-13 signalling and human asthma and atopy.. Hum Mol Genet;.

[34] M Masters M, E Barrett-Connor E. 1985) Parasites and asthma – predictive or protective?. Epidemiology Reviews;.

[35] Marsh DG, Neely JD, Breazeale DR, Ghosh B, Freidhoff LR (1994). Linkage analysis of IL4 and other chromosome 5q31.1 markers and total serum immunoglobulin E concentrations.. Science;.

[36] Maier LM, Howson JM, Walker N, Spickett GP, Jones RW (2006). Association of IL13 with total IgE: evidence against an inverse association of atopy and diabetes.. J Allergy Clin Immunol;.

